# Serum activin A and B, and follistatin in critically ill patients with influenza A(H1N1) infection

**DOI:** 10.1186/1471-2334-14-253

**Published:** 2014-05-10

**Authors:** Rita Linko, Mark P Hedger, Ville Pettilä, Esko Ruokonen, Tero Ala-Kokko, Helen Ludlow, David M de Kretser

**Affiliations:** 1 Intensive Care Unit, Department of Anaesthesia and Intensive Care Medicine, Division of Surgery, Helsinki University Hospital, Helsinki, Finland; 2 Monash Institute of Medical Research, Monash University, Melbourne, Australia; 3 Intensive Care Unit, Kuopio University Hospital, Kuopio, Finland; 4 Departmentof Anaesthesiology, Division of Intensive Care, Oulu University Hospital, Oulu, Finland; 5 Oxford Brookes University, Oxford, UK

**Keywords:** Activin A, Activin B, Follistatin, H1N1 influenza, Acute respiratory distress syndrome, Interleukin 6

## Abstract

**Background:**

Activin A and its binding protein follistatin (FS) are increased in inflammatory disorders and sepsis. Overexpression of activin A in the lung causes similar histopathological changes as acute respiratory distress syndrome (ARDS). ARDS and severe respiratory failure are complications of influenza A(H1N1) infection. Interleukin 6 (IL-6), which in experimental studies increases after activin A release, is known to be related to the severity of H1N1 infection. Our aim was to evaluate the levels of activin A, activin B, FS, IL-6 and IL-10 and their association with the severity of respiratory failure in critically ill H1N1 patients.

**Methods:**

A substudy of a prospective, observational cohort of H1N1 patients in Finnish intensive care units (ICU). Clinical information was recorded during ICU treatment, and serum activin A, activin B, FS, IL-6 and IL-10 were measured at admission to ICU and on days 2 and 7.

**Results:**

Blood samples from 29 patients were analysed. At the time of admission to intensive care unit, elevated serum levels above the normal range for respective age group and sex were observed in 44% for activin A, 57% for activin B, and 39% for FS. In 13 of the 29 patients, serial samples at all time points were available and in these the highest activin A, activin B and FS were above the normal range in 85%, 100% and 46% of the patients, respectively. No difference in baseline or highest activin A or activin B was found in patients with or without acute lung injury (ALI) or ARDS (P > 0.05 for all). Peak levels of IL-6 were significantly elevated in ALI/ARDS patients. Peak activin A and activin A/FS were associated with ventilatory support free-days, severity of acute illness and length of ICU stay (P < 0.05 for all).

**Conclusions:**

Higher than normal values of these proteins were common in patients with H1N1 infection but we found no association with the severity of their respiratory failure.

## Background

Activins are dimeric proteins belonging to the transforming growth factor beta (TGF-β) superfamily. Activin A is involved in many physiological functions including inflammation, tissue remodelling and repair [1-3]. In acute inflammation caused by a lipopolysaccharide (LPS) challenge, serum activin A levels are increased before tumor necrosis factor α (TNFα), interleukin 1β (IL1β) and interleukin 6 (IL6), and stimulate the activin-binding protein, follistatin (FS) which peaks at 3-6 hours post -LPS [4]. Activin has proinflammatory and anti-inflammatory actions [5], and it may modulate the adaptive immune response [6]. Serum activin A is increased in sepsis [7], which is the most common risk factor for acute respiratory distress syndrome (ARDS) [8]. In ARDS patients, activin A levels in bronchoalveolar fluid (BAL) are high, and in mice overexpression of activin A in lungs leads to histopathology resembling human ARDS [9].

Follistatin regulates the activity of activins. Two forms of FS, FS288 and FS315, are products of alternative mRNA splicing. Blocking the action of activin A by FS can halve the mortality of a lethal dose of LPS and highlights the role of activin A in inflammation, leading to therapeutic interest (1). Bleomycin-induced rat lung fibrosis was attenuated [10], and adverse effects of activin A overexpression were decreased by the use of FS in mice [9]. In vivo, blocking activin A enhances adaptive immunity [6].

Influenza A(H1N1) caused a pandemic in year 2009. The majority of H1N1 patients had a mild clinical course, but a minority needed intensive care unit (ICU) treatment for rapidly progressive severe respiratory failure of viral pneumonitis and ARDS [11,12]. Early treatment with antiviral drugs may improve outcome of severe influenza [13], but no specific treatment for ARDS is available. Severe H1N1 infection is associated with hypercytokinemia [14,15] and a dysregulated immune response [16]. The increase in IL-6 has been found to correlate with respiratory involvement in adult [17] and paediatric [18] patients. Further, in ARDS caused by other aetiologies, high IL-6 levels have been associated with worse outcomes [19]. Given these results, any potential therapeutic approach that ameliorates this inflammatory response and disease progression requires evaluation.

Accordingly, our study was carried out to assess the pattern of the novel inflammatory regulators, activins and FS, and to compare them with the classic inflammatory mediators IL-6 and IL-10 in critically ill H1N1 patients. Additionally, we evaluated the association of these markers with the severity of acute illness and respiratory failure in these patients.

## Methods

### Patients and study design

This study was a sub-study of the previously published prospective observational cohort study of consecutive, critically ill influenza A(H1N1) patients during the Finnish H1N1 outbreak from 11 October 2009 to 31 December 2009 [20]. All study patients were confirmed H1N1 positive by real-time reverse polymerase chain reaction (PCR) test. The main study consisted of 132 patients from 23 intensive care units. This sub-study was conducted in four ICUs and all 57 patients of these ICUs were considered eligible for the study.

The ethics committee of Helsinki University Central Hospital approved the study. In the main study informed consent was waived for data collection from patient charts. For this laboratory sub-study signed informed consent was required from the patient or next-of-kin prior to blood sample collection.

We recorded patient demographics and clinical data with an internet-based clinical report form provided by the Finnish Intensive Care Quality Consortium (Intensium Ltd, Kuopio, Finland). The collected data has been described in more detail previously [20]. Briefly, co-morbidities, symptom and medication starting times, ventilatory and other therapies, presence of severe sepsis or septic shock and ARDS [21] were recorded. The degree of hypoxemia was evaluated by partial pressure of arterial oxygen divided by the fraction of inspired oxygen (PaO_2_/FiO_2_).

Age, gender, Simplified Acute Physiology Score (SAPS) II, Sequential Organ Failure Assessment (SOFA) score components, hospital and ICU admission/discharge times, and ICU and hospital mortality were acquired from the routinely collected benchmarking data (Intensium Ltd, Kuopio, Finland).

The blood samples were collected as soon as possible after ICU admission (baseline), and on days 2 and 7. The serum samples were stored at -20°C during the study period, and then transferred and stored at -70°C until analyzed. Serum activin A, activin B, and total FS were determined using specific ELISAs for activin A and B and by radioimmunoassay for follistatin [22-24]. The intra-assay CV for activin A, activin B and FS were 5.7%, 2.7-6.2%, and 5.8%, respectively. The lower levels of detections were as follows: activin A 7.7 pg/mL, activin B 19.0 pg/mL and FS 1.44 ng/mL. Details of the published defined age-related reference values of 138 healthy persons were used for comparison (de Kretser et al. 2013) (Additional file 1). We calculated activin A:FS-ratio (activin A/FS) as an indicator of available activin A activity.

IL-6 levels in serum were measured by a specific human IL-6 ELISA kit BD Biosciences, San Diego, CA using a human recombinant IL-6 preparation supplied with the kit. IL-10 serum levels were also measured using a specific IL-10 ELISA kit, BD Biosciences, San Diego using a human recombinant IL-10 provided with the kit.

### Statistical analysis

Data are presented as median with interquartile range (IQR) or absolute values and percentages as appropriate. We compared the non-parametric data of independent groups using two-tailed Mann–Whitney test and those of several groups using Kruskal-Wallis test. Repeated measurements were compared with Friedman’s test. Due to non-normality of activin A, activin B, and FS, Spearman’s correlation coefficient (r) was used to test relationships. We used SPSS 19 (IBM SPSS Inc, Chicago, IL, USA and GraphPad 6 (GraphPad Software, LaJolla, CA, USA) for statistical analysis.

## Results

Consent for blood sampling was available from 29 out of 57 eligible patients. Demographic data of the study patients compared to H1N1 patients with no consent are presented in Table 1. Median [IQR] of lowest PaO_2_/FiO_2_ was 100 [76–134] mmHg. Positive end-expiratory pressure (PEEP) at admission was 5 [4-8] cmH_2_O in non-invasively ventilated (NIV) patients compared to 8.5 [6.3-12.0] cmH_2_O in invasive ventilation, P < 0.05. No patient in the study was pregnant or in the postpartum state. Oseltamivir and antibiotics treatment and time was recorded in 28 and in 26 of 29 patients, respectively. Corticosteroid medication was given to 19 patients; 9 patients were treated for ARDS with or without shock or airway obstruction and 7 patients for obstruction. Methylprednisolone was given to 17 patients, hydrocortisone to one and 3 patients received both. The median [IQR] duration of corticosteroid therapy during ICU treatment was 3 [0.8] days.

**Table 1:** Characteristics of study patients compared to other patients

	**Included H1N1 patients n** **=** **29**	**Other H1N1 patients ICUs n** **=** **28**	**P value**
Age, years	47 [39–56]	49 [37–55]	0.994
Gender, male	20 (69%)	17 (61%)	0.514
Obstructive pulmonary disease	9 (31%)	8 (29%)	0.839
Chronic cardiac insufficiency	9 (31%)	4 (14%)	0.207
Ischemic cardiac disease	9 (31%)	0	1.000
Body mass index >35 kg/m^2^	11 (39%)	4 (15%)	0.070
SAPS II, points	24 [20-32]	29 [24–39]	0.016
SOFA at 24 h, score	3 [2-7]	6 [3–8]	0.042
SOFA maximum, score	7 [3–9]	6 [4–11]	0.493
Time from symptoms to hospital admission, hours	132 [54–188]	72 [36–168]	0.115
Time from hospital admission to ICU admission	19 [12–33]	19 [8–35]	0.792
CRP (mg/L) at ICU admission	73 [33–163]	57 [26–199]	0.965
PaO_2_/FiO_2_ (mmHg) at ICU admission	135 [101–261]	132 [110–299]	0.492
PaO_2_/FiO_2_ (mmHg), lowest	100 [76–134]	111 [79–269]	0.274
Number of infiltrated quadrants on X-ray at admission	4 [2–4]	2 [1–3]	0.025
ALI or ARDS	19 (66%)	16 (57%)	0.516
Corticosteroid treatment	19 (66%)	13 (46%)	0.2923
Ventilatory support			0.144
No	3 (10%)	6 (21%)	
Non-invasive	13 (45%)	6 (21%)	
Invasive	13 (45%)	16 (57%)	
CPAP or PEEP (mmHg) at admission	7 [5–12]	7 [6–8]	0.485
Length of ventilatory support, days	4 [2–10]	4 [0–9]	0.485
ICU length of stay, days	5 [2–10]	3 [1–9]	0.389
Hospital length of stay, days	12 [7–16]	11 [5–25]	0.812
ICU mortality	1 (83%)	0	1.000
Hospital mortality	1 (3%)	2 (7%)	0.611

SAPS, simplified acute physiology score; SOFA, Sequential Organ Failure Assessment; ICU, intensive care unit; ALI, acute lung injury; ARDS, acute respiratory distress syndrome; CPAP, continuous positive airway pressure; PEEP, positive end-expiratory pressure, median [interquartile range].

**Figure 1:**
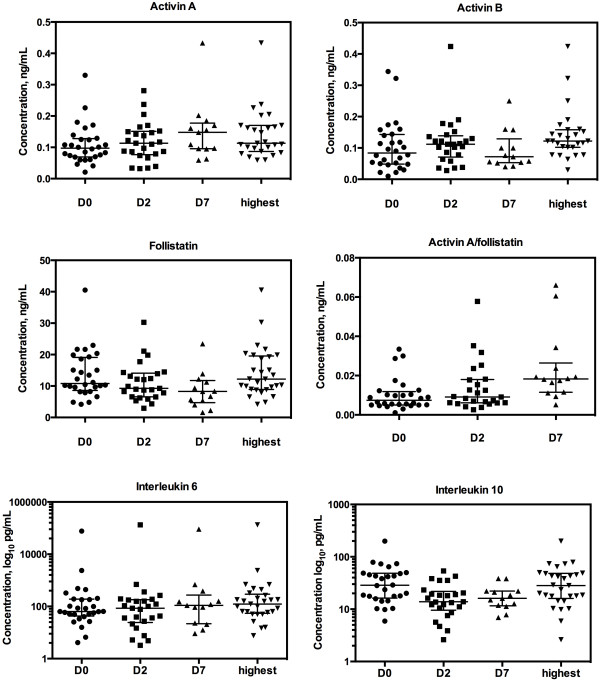
**Serum levels of activin A, activin B, follistatin, the activin A/follistatin ratio, interleukin 6 and interleukin 10 at baseline (D0), and on days two (D2) and seven (D7).** Highest values are also displayed. Markers represent individual patient values, and median with interquartile range.

At baseline the medians [IQR] of serum activin A, activin B and FS were 0.098 [0.069-0.128] ng/mL, 0.084 [0.049-0.143] ng/mL, and 10.80 [8.60-19.12] ng/mL, respectively. Activin A/FS at baseline was 0.0075 [0.0051-0.0118]. At baseline the median [IQR] of IL-6 and IL-10 were 63.93 [42.80-189.91] pg/mL and IL-10 28.60 [16.28-48.26] pg/mL, respectively. Data from other time points are presented in Figure 1.

Of all study patients, serum activin A levels exceeded the upper level of the normal range in 44% of patients at admission (day 1), in 57% on day 2 and 69% on day 7. For serum activin B levels those exceeding the upper limit were 57% on day 1, 76% on day 2 and 54% on day 7. For serum follistatin, those patients exceeding the upper limit of normal were 39% at day 1, 36% on day 2 and 15% at day 7.

Median [IQR] of highest values was 0.110 [0.085-0.168] ng/mL for activin A, 0.122 [0.078-0.155] for activin B, 12.21 [8.90-19.57] for FS. As normal ranges for the IL-6 and IL-10 assays were not available, the data for these cytokines are provided as the IQR for day 1, day 2 and day 7 and for IL-6 on day 1, day 2 and day 7. The IQR for the highest levels of IL6 was 122.91 [54.60-294.55] pg/mL and for IL-10 28.94 [17.78-49.31] pg/mL. On day two IL-6 correlated with activin A (r = 0.664; P = 0.001) and activin B (r = 0.438; P = 0.028) and activin A/FS (r = 0.477; P = 0.016). Day two IL-10 correlated with the respective activin B levels (r = 0.405, P = 0.045). Highest IL-6 correlated with highest activin A (r = 0.599; P = 0.001) and activin A/FS and (r = 0.590; P = .001).

**Figure 2:**
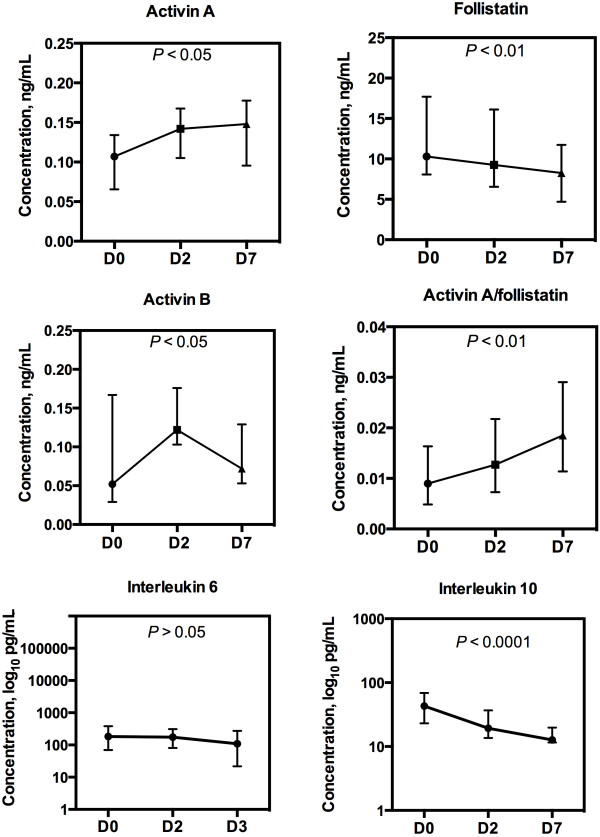
**Activin A, activin B, follistatin, activin A/follistatin, interleukin 6 and interleukin 10 in subset of patients with all serial samples (n = 13) at baseline (D0), and on days two (D2) and seven (D7).** Data presented as median with interquartile range. Comparison made for individual serial data.

Serial measurement data of patients with samples at all time points (n = 13) are presented in Figure 2. The highest activin B level exceeded normal range in all patients. Peak activin A and FS levels were above the normal range in 85% and 46% of patients, respectively.

Baseline activin B, activin A/FS and IL-6 were not associated with oxygenation failure estimated by PaO_2_/FiO_2_ at ICU admission or by lowest PaO_2_/FiO_2_ during ICU stay (Table 2). At baseline, only higher IL-10 was associated with admission oxygenation failure and higher admission positive end-expiratory pressure (PEEP) (P < 0.05 for both), but not with ventilator free days. Lower IL-6 was associated with more ventilator free days, lower organ failure score on the first day and a shorter length of stay (LOS) in ICU (P < 0.05 for all).

**Table 2:** Association of baseline activin A, activin B and activin A/follistatin with severity of respiratory failure

		**Activin A**	**Activin B**	**Activin A/follistatin**
PaO_2_/FiO_2_ at ICU admission	D0	r = -0.093; P = 0.659	r = -0.073; P = 0.730	r = -0.023; P = 0.914
	highest	r = -0.181; P = 0.385	r = 0.124; P = 0.556	r = -0.132; P = 0.529
PaO_2_/FiO_2_ lowest during ICU stay	D0	r = 0.122; P = 0.538	r = 0.186; P = 0.343	r = 0.105; P = 0.596
	highest	r = -1.31; P = 0.498	r = 0.052; P = 0.719	r = -0.117; P = 0.547
PEEP at admission	D0	r = -0.071; P = 0.773	r = -0.093; P = 0.706	r = -0.039; P = 0.875
	highest	r = 0.297; P = 0.217	r = 0.118; P = 0.629	r = 0.102; P = 0.678
Ventilatory support free days	D0	r = -0.143; P = 0.467	r = 0.100; P = 0.613	r = -0.068; P = 0.729
	highest	r = -0.452; P = 0.014	r = -0.166; P = 0.391	r = -0.446.; P = 0.015
SOFA score, at 24 hours	D0	r = 0.351; P = 0.067	r = 0.047; P = 0.814	r = 0.222; P = 0.257
	highest	r = 0.466; P = 0.011	r = -0.095; P = 0.624	r = 0.369; P = 0.049
SAPSII score	D0	r = 0.252; P = 0.196	r = -0.020; P = 0.921	r = 0.191; P = 0.330
	highest	r = 0.434; P = 0.019	r = -0.065; P = 0.739	r = 0.471; P = 0.010
C-reactive protein at ICU admission	D0	r = 0.131; P = 0.516	r = 0.140; P = 0.485	r = 0.057; P = 0.778
	highest	r = 0.232; P = 0.235	r = 0.279; P = 0151	r = 0.201; P = 0.305
Length of ICU stay	D0	r = 0.202; P = 0.303	r = -0.077; P = 0.698	r = 0.093; P = 0.638
	highest	r = 0.422; P = 0.023	r = -0.128; P = 0.507	r = 0.409; P = 0.028

ICU, intensive care unit; PEEP, positive end-expiratory pressure; SOFA, Sequential Organ Failure Assessment; SAPS, Simplified Acute Physiology Score; r, Spearman’s correlation coefficient; D0, baseline.

The highest activin A and activin A/FS were associated with less ventilator free days, higher acute disease severity and longer length of stay in ICU, but not with admission PEEP (Table 2). Peak IL-6 and IL-10 were associated with higher admission levels of PEEP (P < 0.05), less ventilator free days, higher acute disease severity and longer stays in ICU.

**Figure 3:**
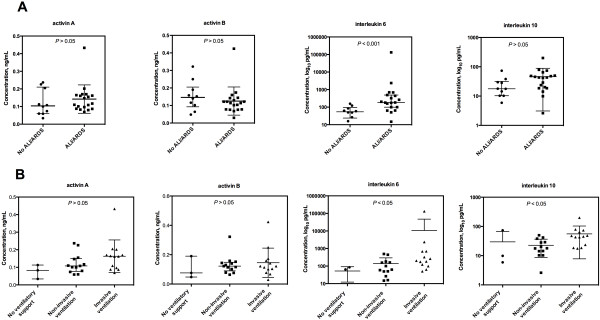
**Peak activin A, activin B, interleukin 6 and interleukin 10 according to ALI/ARDS (A) and type of ventilatory support (B).** Markers represent highest individual patient values, and median with interquartile range. Comparison made between all groups.

No difference in the baseline or peak activin A and activin B levels regarding ALI/ARDS or type of ventilatory support were found (Figure 3). At ICU admission, IL-6 and IL-10 were higher in patients with ALI/ARDS (P < 0.01 for both), and IL-10 was higher in patients needing invasive ventilation (P < 0.05) on ICU admission. The peak IL-6 level was higher in ALI/ARDS, and IL-6 and IL-10 were higher in invasively ventilated patients (Figure 3).

## Discussion

This study is the first report of elevated serum levels of activin A, activin B and FS in critically ill patients with H1N1 infection. In serial measurements activin A, activin A/FS increased, FS and IL-10 decreased, and IL-6 remained at the admission level during the first week of ICU treatment.

Increase of activin A and FS is in accordance with previous findings in an LPS induced experimental sepsis model [1] and in septic patients [7]. In septic patients, as in the patients in our study, the levels of serum activin A tended to be elevated for days. This finding differed from the rapid peak (1 hour) and return to basal level (5 hours) after experimental LPS injection [5]. In the LPS-induced sepsis model, the higher serum level of activin A was associated with a fatal outcome [1], but not in a small study of septic patients [7]. The small sample size and low mortality in our study prevented any mortality analysis.

To our knowledge, activin B levels have not been reported in critically ill patients. This study showed elevated activin B levels in most of the patients, but the trend was different compared to activin A, suggesting different regulatory mechanisms. In part this is reflected in the acute response to LPS since the activin A response is rapid and does not require new protein synthesis whereas the activin B response is delayed and dependent on new protein synthesis (Hedger et al. unpublished data). This novel finding indicates the need for further studies to delineate the role of activin B in inflammation and fibrosis. To date, activin B, with an amino acid sequence homology to activin A of 65% has been considered as a less potent member of this family of proteins as it was only partially able to compensate for the absence of activin A in a mice with deletion of the activin A subunit [25].

In animal models, FS peaks (4 hours) after activin A and stays elevated longer (24 hours). This pattern arises from the capacity of activin A to stimulate FS production [1]. As the trend of activin A was increasing during the first 7 days, the trend of FS was decreasing. The increasing activin A/FS, an indicator of available activin activity, may implicate dysregulation of activin A and a consequent lung injury. Strong expression of activin A has been found on alveolar macrophages in diffuse alveolar damage, lung fibrosis, and in pulmonary arteries in pulmonary hypertension [2], all of which findings may be present in ARDS. Furthermore, the rapid increase in lung activin concentrations in mice given LPS has been shown to be due in part to the rapid movement of neutrophils into the lungs and the presence of activin A in their secretory granules [26]. Furthermore, TNFα has the capacity to release activin A from neutrophil secretory granules [27]. Recently, Apostolou and colleagues demonstrated that histopathology in ARDS was associated with overexpression of activin A in mice lungs and, additionally, activin A was elevated in BAL fluid in ARDS patients [9].

Of note, contrary to expectations, we found no association between circulating activin A, FS or activin A/FS and ALI/ARDS. There may be several explanations for this result. First, the evidence of parallel circulating activin A and expression in lungs and/or BAL fluid in patients is still lacking. The degree of oxygenation failure in our patients was severe and, thus, obtaining BAL samples, unnecessary to patient treatment, was not justified. Second, compared to animal studies, patient cohorts are more heterogeneous. Although, in this study, all patients were confirmed to have the same infection, other standardization was not possible and different co-morbid conditions, obesity, and septic bacterial secondary infections were present. Third, time from the start of symptoms to hospitalisation and ICU treatment was relatively short, and thus, prompt treatment with oseltamivir and antibiotics may have influenced disease severity and outcome. In addition, the more severely ill patients were more likely to have been treated with corticosteroids [28]. Corticosteroids suppress systemic inflammation in ARDS patients [29], and together with a neuraminidase inhibitor may attenuate tissue damage during experimental influenza infection [30]. Further, administration of glucocorticoids also suppresses activin A expression [31,32]. Mechanical ventilation [33], prone position [34], and neuromuscular blocking agents [35] all may affect the inflammatory response. Finally, although we analyzed all available samples from cohort of patients with H1N1, due to required informed consent, the patients were not consecutive and the sample size was relatively small. This may have caused a type II error in detecting existing differences in activin and FS levels with regard to disease severity.

In accordance with the previous studies in ARDS [19], we found higher IL-6 in ALI/ARDS patients. Our results also corroborate with previous studies showing an IL-6 increase with respiratory severity in H1N1 influenza [14,15,17,18]. Interestingly, the evolution of IL-6, IL-10 and activin A were different over time. Activin A, activin B and activin A/FS correlated with IL-6 only on day two.

The importance of measuring activin A and B in critically ill patients was illustrated by our previous study of patients in ICU with acute respiratory failure [36]. That study established that patients with both serum activin A and B above the normal range had the highest mortality levels. This outcome may be associated with the capacity of increased levels of activin A to cause apoptosis of hepatocytes and B lymphocytes and to cause the production of nitric oxide (reviewed in [37]). Further exploration of the capacity of soluble activin receptor blockers and follistatin as agents capable of regulating the bioactivity of the activins and their possible role in the treatment of such patients may be of value in the future.

To our knowledge, this is the first study to report increased levels of activin A, activin B and FS, compared to normal range, and simultaneous IL-levels in severe H1N1 influenza. Regrettably, the inclusion of only critically ill patients prevented evaluation of potential association between these inflammatory markers and disease severity, which needs to be scrutinized in a larger population of critically ill patients.

## Conclusions

This study establishes that in critically ill patients with severe H1N1 Influenza, the levels of activins A, B and follistatin are elevated in a significant proportion of patients. However, the magnitude of the increase did not correlate with the severity of the infection and the degree of respiratory failure.

## Abbreviations

ICU: Intensive care unit; ALI: Acute lung injury; ARDS: Acute respiratory distress syndrome; FS: Follistatin; activin A/FS activin A: Follistatin-ratio; IL-6: Interleukin 6; IL-10: Interleukin 10; PaO2/FiO2: Partial pressure of oxygen divided by the fraction of inspired oxygen; PEEP: Positive end-expiratory pressure; LOS: Of stay; SOFA: Sequential organ failure assessment; SAPS: Simplified acute physiology score.

## Competing interests

RL, VP, ER, TAK, HL and MPH declare no conflict of interest. DdeK is a director of Paranta Biosciences, a company developing follistatin as a therapeutic and with MPH hold shares in the company recognizing their intellectual input into the provisional patents, licensed to Paranta.

## Authors’ contributions

RL, ER and TAK were involved in planning, data collection and reporting the original study. RL analyzed the data, made the statistical analysis and drafted the manuscript. VP was involved in planning the original study. VP, MPH and DdeK were responsible for planning this substudy, and contributed in data analysis and manuscript preparation. HL developed the activin B assay reagents. ER, TAK and HL participated in drafting and revision of the manuscript. All authors have read and approved the final manuscript.

## Pre-publication history

The pre-publication history for this paper can be accessed here:

http://www.biomedcentral.com/1471-2334/14/253/prepub

## Supplementary Material

Additional file 1 Reference ranges for activin A, activin B and follistatin, presented as mean ± SEM concentrations and 95% C.I. M = male, F = female. Click here for file

## The Finnish H1N1 study group: participating hospitals and study investigators

Satakunta Central Hospital, Dr. Vesa Lund; East Savo Central Hospital, Dr. Markku Suvela; Central Finland Central Hospital, Dr. Raili Laru-Sompa; South Savo Central Hospital, Dr. Heikki Laine; North Karelia Central Hospital, Dr. Matti Reinikainen; Seinäjoki Central Hospital, Dr. Kari Saarinen; South Karelia Central Hospital, Dr. Seppo Hovilehto; Päijät-Häme Central Hospital, Dr. Pekka Loisa; Vaasa Central Hospital, Dr. Raku Hautamäki; Kanta-Häme Central Hospital, Dr. Ari Alaspää; Lappi Central Hospital, Dr. Outi Kiviniemi; Keski-Pohjanmaa Central Hospital, Dr. Tadeusz Kaminski; Kymenlaakso Central Hospital, Dr. Seija Alila, Dr. Jussi Pentti; Helsinki University Hospital, Jorvi, Peijas and Meilahti Hospitals, Dr. Tero Varpula, Dr. Anne Kuitunen, Dt. Tuomas Oksanen, Dr. Timo Suonsyrjä; Turku University Hospital, Dr. Juha Perttilä; Tampere University Hospital, Dr. Sari Karlsson, Dr. Jyrki Tenhunen; Länsi-Pohja’s Central Hospital, Dr. Jorma Heikkinen; Kuopio University Hospital, Dr. Esko Ruokonen, Ilkka Parviainen; Oulu University Hospital, Dr. Tero Ala-Kokko, Dr. Jouko Laurila; Turku University Hospital, Paediatric ICU, Dr. Janne Kataja; Helsinki University Hospital, ICU of Hospital for Children and Adolescents, Dr. Paula Rautiainen.

## Key messages

- Activin A and B levels were elevated in most of the H1N1 patients

- The baseline levels of Activin A and B did not correlate with the severity of respiratory symptoms in critically ill influenza A(H1N1) patients, but highest activin A and activin A/FS levels correlated with less ventilator free days, increased acute disease severity and increased ICU LOS

- Future studies are justified to explore the role of acitivins and follistatin in ARDS.
