# Supplementary material for: Serum activin A and B, and follistatin in critically ill patients with influenza A(H1N1) infection
Source: BMC Infect Dis. 2014 May 10;14:253. doi: 10.1186/1471-2334-14-253 (PMC4101860; doi:10.1186/1471-2334-14-253)
Supplement: Additional file 1 — Reference ranges for activin A, activin B and follistatin, presented as mean ± SEM concentrations and 95% C.I. M = male, F = female. [file 1471-2334-14-253-S1.docx]

Additional File 1: Linko et al

Table. Reference ranges for activin A, activin B and follistatin, presented as mean ± SEM concentrations and 95% C.I. M = male, F = female

| Analyte | Sex | Age (years) | n | Concentration (ng/mL) | 95% C.I. |
| --- | --- | --- | --- | --- | --- |
| Activin A | M/F | 18-50 | 56 | 0.087±0.004 | 0.080-0.095 |
|  |  | 51-65 | 52 | 0.110 ±0.005 | 0.099-0.120 |
|  |  | ≥66 | 30 | 0.150 ± 0.009 | 0.130-0.170 |
| Activin B | F | 18-50 | 29 | 0.085±0.005 | 0.074-0.096 |
|  |  | 51-65 | 31 | 0.062±0.005 | 0.051-0.073 |
|  |  | ≥66 | 20 | 0.071±0.005 | 0.060-0.082 |
| Activin B | M | 18-50 | 27 | 0.061±0.004 | 0.053-0.069 |
|  |  | 51-65 | 21 | 0.064±0.005 | 0.053-0.074 |
|  |  | ≥66 | 10 | 0.086±0.008 | 0.069-0.100 |
| Follistatin | M/F | 18-50 | 56 | 11.67±0.63 | 10.40-12.94 |
|  |  | 51-65 | 52 | 12.61±0.58 | 11.44-13.78 |
|  |  | ≥66 | 30 | 14.36±0.71 | 12.90-15.82 |
